# Genetic background determines response to hemostasis and thrombosis

**DOI:** 10.1186/1471-2326-6-6

**Published:** 2006-10-05

**Authors:** Jane Hoover-Plow, Aleksey Shchurin, Erika Hart, Jingfeng Sha, Annie E Hill, Jonathan B Singer, Joseph H Nadeau

**Affiliations:** 1 Department of Cardiovascular Medicine, Joseph J. Jacobs Center for Thrombosis and Vascular Biology, Department of Molecular Cardiology, Cleveland Clinic Lerner Research Institute, Cleveland, Ohio, USA; 2 Department of Genetics, Case University School of Medicine, Cleveland, Ohio, USA; 3 Novartis Institutes for Biomedical Research, Cambridge, Massachusetts, USA

## Abstract

**Background:**

Thrombosis is the fatal and disabling consequence of cardiovascular diseases, the leading cause of mortality and morbidity in Western countries. Two inbred mouse strains, C57BL/6J and A/J, have marked differences in susceptibility to obesity, atherosclerosis, and vessel remodeling. However, it is unclear how these diverse genetic backgrounds influence pathways known to regulate thrombosis and hemostasis. The objective of this study was to evaluate thrombosis and hemostasis in these two inbred strains and determine the phenotypic response of A/J chromosomes in the C57BL/6J background.

**Methods:**

A/J and C57Bl/6J mice were evaluated for differences in thrombosis and hemostasis. A thrombus was induced in the carotid artery by application of the exposed carotid to ferric chloride and blood flow measured until the vessel occluded. Bleeding and rebleeding times, as surrogate markers for thrombosis and hemostasis, were determined after clipping the tail and placing in warm saline. Twenty-one chromosome substitution strains, A/J chromosomes in a C57BL/6J background, were screened for response to the tail bleeding assay.

**Results:**

Thrombus occlusion time was markedly decreased in the A/J mice compared to C57BL/6J mice. Tail bleeding time was similar in the two strains, but rebleeding time was markedly increased in the A/J mice compared to C57BL/6J mice. Coagulation times and tail morphology were similar, but tail collagen content was higher in A/J than C57BL/6J mice. Three chromosome substitution strains, B6-Chr5^A/J^, B6-Chr11^A/J^, and B6-Chr17^A/J^, were identified with increased rebleeding time, a phenotype similar to A/J mice. Mice heterosomic for chromosomes 5 or 17 had rebleeding times similar to C57BL/6J mice, but when these two chromosome substitution strains, B6-Chr5^A/J ^and B6-Chr17^A/J^, were crossed, the A/J phenotype was restored in these doubly heterosomic progeny.

**Conclusion:**

These results indicate that susceptibility to arterial thrombosis and haemostasis is remarkably different in C57BL/and A/J mice. Three A/J chromosome substitution strains were identified that expressed a phenotype similar to A/J for rebleeding, the C57Bl/6J background could modify the A/J phenotype, and the combination of two A/J QTL could restore the phenotype. The diverse genetic backgrounds and differences in response to vascular injury induced thrombosis and the tail bleeding assay, suggest the potential for identifying novel genetic determinants of thrombotic risk.

## Background

Family history [1] is the strongest risk factor for cardiovascular diseases (CVD). While a number of genetic mutations have been identified, these account for only a small percentage of the CVD in human populations. Thrombus formation on fissured atherosclerotic plaques is the precipitating event in the transition from a stable or subclinical atherosclerotic disease and leads to acute myocardial infarction, ischemic stroke or peripheral arterial occlusion. Arterial and venous thrombosis are complex responses and are influenced by multiple genetic and environmental factors [2-5]. Polymorphisms and mutations in coagulation factors, fibrinolytic factors, platelet surface receptors, methylenetetrahydrofalate reductase, endothelial nitric oxide synthase, and antioxidant enzymes have been implicated as genetic determinants of susceptibility to thrombosis [6,7]. Great strides have been made in the diagnosis and treatment of thrombosis in the last decade. However, strategies to prevent thrombosis have lagged far behind due, in part, to the contribution of multiple, and as yet undefined, genetic factors that lead to thrombotic risk. Moreover, it remains unclear how genetic background influences the function of molecules and pathways known to regulate thrombus formation and lysis and, thereby, contributes to the risk of thrombotic disease.

Many of the available inbred mouse strains, such as C57BL/6J (B6) and A/J, have been classified as resistant or susceptible to particular complex diseases (Table 1). These two strains have diverse responses to diet-induced obesity [8] (B6 susceptible; A/J resistant), diet-induced atherosclerosis (B6 susceptible; A/J resistant) [9], arterial ligation-induced neointimal hyperplasia (B6 resistant; A/J resistant), and ligation induced vessel remodeling (B6 resistant; A/J susceptible) [10]. These conditions are predisposing to thrombosis per se, but these mice have not been systematically evaluated for thrombosis. Clot formation and lysis, which ultimately determine thrombosis, requires platelet aggregation, coagulation, and fibrinolytic functions. Interactions among these pathways may occur and additional factors may modulate these processes. Identification of novel modulators and factors of these pathways can be identified by associating the phenotype with a location(s) on a specific chromosome, a quantitative trait locus (QTL).

**Table 1:** Identification of mice susceptible or resistant to obesity, atherosclerosis, and vascular injury

**Mouse Strain/Genotype**	**Diet-induced Obesity**	**Diet-induced Atherosclerosis**	**Neointimal Hyperplasia**	**Vessel Remodeling**
C57BL/6J (B6)	S [8]	S [9]	R [10]	R [10]
A/J	R [8]	R [9]	R [10]	S [10]
Lep^ob^	S [41]	S [42]	R [43]	R [43]
Plg-/-	R [44]	S (apoE-/-) [45] R (transplant) [46]	R [47,48]	R [48]
PAI-1-/-	S [18]	R [49,50]	R [50]	R [50]

R: resistant; S: susceptible. Reference provided by number in parenthesis. Lep^ob^, Plg-/-, and PAI-F1-/- mice were in a B6 background.

A panel of chromosome substitution strains (CSS) was generated [11] by a "marker-assisted" breeding program where the progeny of a B6 × A/J cross were successively backcrossed to the B6 mice. Genetic markers were used to identify homozygosity in the background (B6) and the individual A/J chromosome. These strains have one chromosome from A/J mice in a B6 background. This allows the identification of a trait in one or more CSS and implies that at least one QTL resides on this chromosome. Use of this panel requires fewer mice to determine the QTL than does a genome-wide scan. Another advantage is the ability for detection of QTL in the presence of other QTL. In addition, initial screens with the CSS simplify the fine-mapping of QTL. Several studies of this CSS panel, B6 Chr1-19, X, Y^A/J^, have been reported for behavior [12], weight gain [13], sterols [13], and plasma amino acids levels [13,14], and have identified many more QTL than studies of the same traits using a genome-wide scan.

The purpose of this study was to evaluate the two inbred strains of mice for prothrombotic risk, utilizing a ferric chloride vascular injury model, tail bleeding assay, and measures of coagulation and fibrinolysis. The results of this study indicate that the two inbred strains, B6 and A/J mice, have diverse prothrombotic phenotypes, unrelated to coagulation or platelet aggregation. In addition, in the CSS, expression of the A/J phenotypes, rebleeding time and arterial occlusion, was modified in the B6 background, and suggested that interactions occurred among the A/J QTL. Thus, it should be possible to identify independent genetic determinants of susceptibility to pathological haemostasis and thrombosis.

## Methods

### Mice

The inbred strains, B6 (#000664), Lep^ob ^(#000632), A/J (#000646) and gene-targeted plasminogen (Plg) activator inhibitor deficient mice (PAI-1-/-) (#002507) [15] mice in a B6 background were obtained from Jackson Laboratory (Bar Harbor, Maine) at 6 wks-of-age. The Plg-/- mice were generated as previously described [16] and maintained in the B6 background, generated by crossing mice from the original mixed (B6:129) background for eight generations with B6 mice. The Plg-/- mice do not reproduce well and Plg heterozygous pairs were used for breeding. Genotypes (Plg+/+, Plg+/-, Plg-/-) of the offspring were determined by a PCR assay at 3–4 weeks-of-age from an ear punch [17]. Breeding pairs of the CSS mice were transferred from Dr. Nadeau's laboratory. All mice were housed and bred in the Biological Resource Unit at the Cleveland Clinic Foundation (CCF). Mice were housed in sterilized isolator cages, maintained on a 14 hr light/10 hr dark cycle, and were provided sterilized food and water ad libitum. Mice were fed a standard autoclavable laboratory diet consisting of: 23% protein, 4.5% fat, 6% fiber, (Purina, St. Louis, MO), and tested between 7 and 9 wks-of-age. All animal experiments were performed in accordance with a protocol approved by the Institutional Animal Care and Research Advisory Committee at CCF.

### Vascular injury

To induce thrombosis formation in the carotid artery, a ferric chloride (FeCl_3_) model of vessel injury [18,19] was employed. Mice were anesthetized with ketamine/xylazine (80 mg/kg, 5 mg/kg), a midline cervical incision was made and the left common carotid artery isolated by blunt dissection. The flow probe (Transonic Systems, model 0.5PSB) was placed under the artery and when a stable baseline was reached, a 0.5 × 2 mm strip of filter paper saturated with 10% FeCl_3 _solution was applied to the surface of the carotid artery for 3 min. Occlusion time was determined from the addition of the FeCl_3 _solution to the occlusion of the artery (minimum blood flow). The flow probe was in place from the establishment of the baseline until several min after the stable occlusion had been reached or stopped at 30 min if it had not occluded. Blood flow was recorded every 10 sec (Transonic Systems, model TS420). There was no difference in body weight among the mice that were tested in the vascular injury model.

### Bleeding and rebleeding assay

For the tail bleeding assay, mice were anesthetized with ketamine/xylazine (80 mg/kg, 5 mg/kg), the tail prewarmed for 5 min in 10 mL of saline at 37°C in a water bath. The tail was lifted from the saline and a 5 mm tail segment amputated and immediately returned to the saline. Bleeding time was measured as the time between the start of bleeding to cessation of bleeding. With the tail remaining in the same saline solution, the rebleeding time was measured from the time between bleeding cessation and the start of the second bleeding.

### Coagulation, Plg, α_2_-antiplasmin, fibrinogen, fibrinolytic and PAI-1 assays

Mice were anesthetized with Isoflurane (Abbott), bled from the orbital sinus into uncoated capillary tubes, and a drop of blood immediately placed on a reagent strip (Synbiotics) for prothrombin time (PT) or activated partial thromboplastin time (aPTT) determinations and inserted into the precalibrated Coagulation Analyzer (SCA2000, Synbiotics). PAI-1 activity was determined according to the method of Chandler *et al *[20]. PAI-1 antigen concentration was determined by ELISA [21] using sheep-anti mouse PAI-1 (American Diagnostica) as the capture antibody and mouse PAI-1 (American Diagnostica) as the standard (0–3.5 ng/mL). Plg was determined with a chromogenic assay [22] and the fibrinolytic activity determined by fibrin degradation [23]. For the negative controls in these assays, plasma from PAI-1 or Plg deficient mice was used. Functionally active mouse α_2_-antiplasmin was determined with a plasmin capture assay and detection with a mouse antibody (Molecular Innovations, Southfield, MI), and fibrinogen with an ELISA assay with antibodies that recognize mouse fibrinogen (Hyphen BioMed, France).

### Histochemical and immunohistochemistry analysis

The frozen tails or injured carotids in OCT were sectioned at 5 μm thickness and stained with Masson's Trichrome (HT 15, Sigma Diagnostics). For quantitative analysis of collagen area in the tail sections, four sections from 2–3 sites were measured using computer assisted image analysis (Image-Pro Plus, Cybernetics). For the area determination of the injured carotid lumen, 3–4 sections from different sites approximately 100 μm apart were analyzed and averaged for each mouse.

### Statistics

Data are presented as mean ± SEM. The statistical analysis for comparisons between A/J and B6 mice and between wild-type (WT) and Plg-/- littermates was compared with a two-tailed t-test. Differences among B6, Lep^ob^, PAI-1-/- mice were determined by a one-way ANOVA and a Newman-Keuls post-test. A value of *P *< 0.05 was considered significant. The statistical analysis for comparisons between B6 mice and CSS were determined with two-tail t-tests and the level of the significant *P*-values (see figure legends) determined with a Bonferroni correction to account for multihypothesis testing [13].

## Results

### Marked differences in arterial thrombus formation in B6 and A/J mice

**Figure 1:**
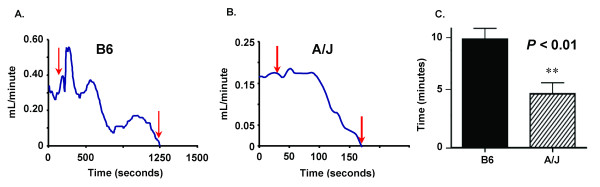
**Arterial occlusion time in B6 and A/J mice**. **Panels A and B **are representative occlusion curves from each genotype. Red arrows indicate duration of ferric chloride application to the carotid. **Panel A: **B6 mice, **Panel B: **A/J mice. **Panel C: **Arterial occlusion time, mean ± SEM of 14–17 mice per genotype. Symbol indicates a significant difference (**P < 0.01) between B6 mice determined with a t-test.

The FeCl_3 _cartoid artery injury model [24], a well-characterized arterial thrombosis model, was used to induce an occlusive thrombus in the B6 and A/J mouse strains under investigation. A marked difference in arterial thrombus occlusion time was found between the B6 and the A/J mice (Figure 1A). The occlusion time in the A/J mice was 2-fold less than for the B6 mice. A 5% dose of FeCl_3 _was tested in the A/J and B6 mice and the occlusion time was lower in the A/J than the B6 mice, the same results as with the 10% dose. The occlusion time (min for both B6 (15 ± 3, n = 3) and A/J (8 ± 1, n = 3) at 5% were longer than at 10% for B6 (10 ± 1, n = 17 and A/J (5 ± 2, n = 14). The curves of blood flow after FeCl_3 _application indicated a pattern with a gradual decrease before occlusion for the B6 mice (Figure 1B), but for the A/J mice the blood flow decreased abruptly to zero (Figure 1C), demonstrating a marked difference from the B6 strain. The mean occlusion times were significantly different between the two strains (Figure 1A). The size of the carotids in the two strains was noticeably different. The area (mm^2^) of the lumen (0.066 ± 0.007, n = 6) was significantly less in the A/J mice than for B6 mice (0.128 ± 0.007, n = 6). However, the ratio of the thrombus to lumen was similar for A/J (0.63 ± 0.03) compared to B6 mice (0.67 ± 0.09). Calculated sheer stress rates [25] were 14% less in the A/J mice when compared to B6 mice.

### Marked differences in rebleeding time in B6 and A/J mice

**Figure 2:**
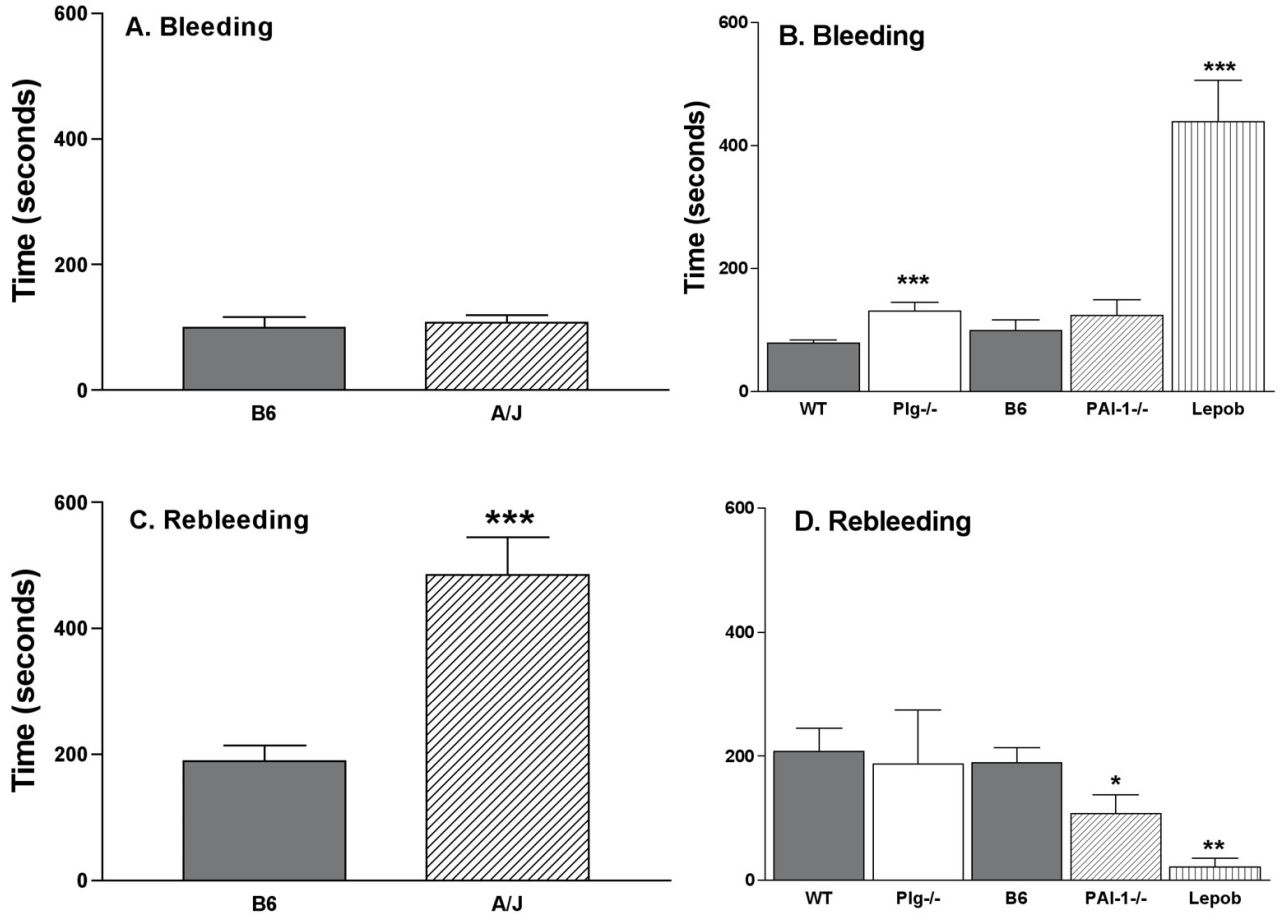
**Bleeding and rebleeding time in B6, A/J, Lep**^ob^**, Plg-/- and PAI-1-/- mice**. **Panel A, B: **Bleeding time (seconds) and **Panel C, D: **Rebleeding time (seconds). To determine statistical differences, values from B6 and A/J and from WT and Plg-/- littermates were compared with a t-test, and values from B6, PAI-1-/- and Lep^ob ^were compared by a one-way ANOVA and a Newman-Keuls post-test. Symbols indicate a significant difference (*P < 0.05, **P < 0.01, ***P < 0.001) between B6 or WT mice. Values are the mean ± SEM of 10–34 mice per genotype.

The bleeding time, an indicator of hemostatic activity, was determined after prewarming the tail, clipping a 5 mm segment, placing the tail in warm saline and measuring the time for the bleeding to stop. We found this procedure was consistent and reproducible in different mouse strains and was not gender dependent. Bleeding time was not different in A/J mice compared to B6 mice (Figure 2A). In order to determine if specific pathways were altered in the B6 and A/J strains, mice with known alterations in hemostasis, Lep^ob ^mice, or thrombosis, Plg-/- and PAI-/- mice, were also tested. The Lep^ob ^mice have impaired platelet aggregation [26] and were evaluated as a reference for platelet function. In the Lep^ob ^mice (Figure 2A) there was nearly a 10-fold increase in bleeding time compared to B6 mice. Bleeding time was also significantly (P = 0.0001) increased by 66% in the Plg-/- mice compared to WT mice littermates. Bleeding time was not different in PAI-1-/- mice compared to B6 mice. In contrast to the Lep^ob ^mice with a platelet defect and 10-fold increase in bleeding time, there was no difference in the bleeding time between B6 and A/J mice and suggested platelet aggregation is not altered in these two strains.

Rebleeding time, an indicator of thrombus stability, was measured as the time between the cessation of bleeding and the start of the second bleeding (Figure 2B). Rebleeding times were significantly higher in A/J (2.6-fold, *P *< 0.001) mice than the B6 mice. In contrast, the rebleeding time was nearly 8.5-fold less in the Lep^ob ^mice than the B6 mice. To determine how alterations in the fibrinolytic system may affect rebleeding time, Plg-/- and PAI-1-/- mice were also tested (Figure 2B). Rebleeding time was not different in Plg-/- mice compared to WT littermates, but PAI-1-/- mice had a significantly (*P *< 0.05) reduced rebleeding time, which was 1.8-fold lower than for the B6 mice. Although a Plg deficiency did not result in a prolonged rebleeding time as anticipated, a PAI-1 deficiency resulted in a shortened time. The markedly increased rebleeding time in the A/J may suggest differences in the regulation of the Plg network.

### No difference in blood coagulation between B6 and A/J mice

To determine if differences in coagulation were contributing factors to the bleeding and rebleeding results, prothrombin time (PT) and activated partial thromboplastin time (aPTT) were measured. PT is a measurement of the extrinsic coagulation pathway (activation of Factor VII by tissue factor) while aPTT is a measurement of the intrinsic pathway (activation of Factors XII, XI, IX and VIII). The products of both pathways activate Factor X, which initiates the common pathway leading to thrombin generation and fibrin clot formation. PT and aPTT were measured in the B6 and A/J mice and values were not different between the two mouse strains (Table 2). These results suggest that the coagulation pathway is not different in the two inbred strains.

**Table 2:** Coagulation and Plasminogen System Parameters in B6 and A/J inbred mouse strains

	**B6**	**A/J**
Prothrombin Time (sec)	17 ± 1	18 ± 1
aPTT Time (sec)	66 ± 3	61 ± 7
Plasminogen (μg/mL)	131 ± 5	176 ± 9**
Fibrinolytic Activity (nM)	2.6 ± 0.1	3.0 ± 0.1*
PAI-1 Antigen (ng/mL)	7.4 ± 0.7	7.5 ± 0.7
PAI-1 Activity (U/mL)	185 ± 10	229 ± 8**
α_2_-antiplasmin (μg/mL)	170 ± 6	195 ± 5**
Fibrinogen (ng/mL)	1.6 ± 0.04	1.7 ± 0.05*

Values are the mean ± SEM. *P < 0.05, **P < 0.01. n = 5–8.

### Plg and PAI-1 higher in A/J than B6 mice

Plg concentration was 34% higher in the A/J mice than in B6 mice. In addition, fibrinolytic activity, PAI-1 activity, and α_2_-anitplasmin were also increased in the A/J mice when compared to the B6 mice (Table 2). The functional consequences of these differences are not clear, since fibrinogen is also higher in the A/J mice. To determine whether there were protein structure changes of Plg from the A/J mice, plasma from the B6 and the A/J mice were subjected to electrophoresis under reduced, non-reduced, and non-denaturing conditions. No difference in migration for immunostained Plg under these conditions was observed (data not shown). The density of the Western blot from SDS-PAGE under reducing conditions of plasma of varying volumes from B6 and A/J mice was compared and the density of the bands was 56% higher in the A/J than the B6 mice (six individual mice were tested on at least two occasions) from the same amount of plasma (Fig. 1B). Thus, the concentration of Plg in plasma from the A/J mice was higher than plasma from the B6 mice as determined by two methods and no difference in migration after electrophoresis was observed, suggesting no marked changes in the structure of Plg in the two mouse strains.

### Tail morphology in B6 and A/J

**Figure 3:**
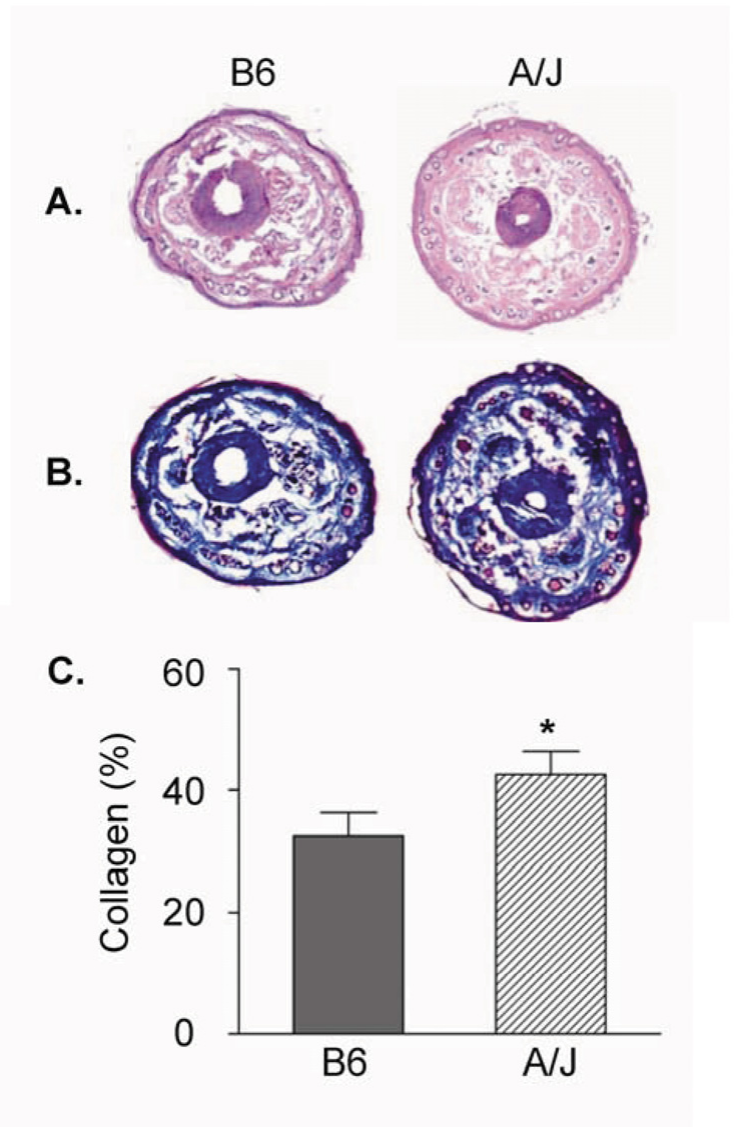
**Tail morphology and collagen**. Tails were excised, embedded in OCT, sectioned, and stained with Hematoxylin/Eosin or Masson's Trichrome. **Panel A: **Representative sections stained with hematoxylin and eosin from B6 and A/J mice. **Panel B: **Representative sections stained with Masson's Trichrome from B6 and A/J mice. The blue color identifies the collagen. **Panel C: **The percentage of the collagen area (excluding center bone/cartilage) of the total tail area of each section was determined in 3–4 sections at 2–3 sites for each tail and averaged for each mouse. Values are the mean ± SEM of 4–8 mice per genotype. Symbol indicates a significant difference (*P < 0.05) between B6 mice determined by t-test.

The tails of the mice were examined for differences that might account for the variations in the bleeding and rebleeding values. Sections of the tail from the B6 and A/J mice were stained with Hematoxylin/Eosin or Masson's Trichrome and representative sections are shown in Figure 3. No obvious qualitative differences were observed in hair follicles, sebaceous glands, or center bone/cartilage in Hematoxylin/Eosin (Figure 3A) stained sections. Sections of the tails were stained with Masson's Trichrome (Figure 3B) that detects collagen, the major platelet adhesive substratum for initiation of thrombus formation. Collagen content (% tail area) was quantified (Figure 3C) from 3–4 sections from 2–3 areas/mouse. The amount of collagen was greater in the A/J than the B6 mice (P < 0.05). Collagen in vessels in adipose tissue in the A/J mice is also increased compared to the B6 mice (data not shown).

### Tail bleeding assay identifies three chromosomes with QTL for rebleeding in CSS

**Figure 4:**
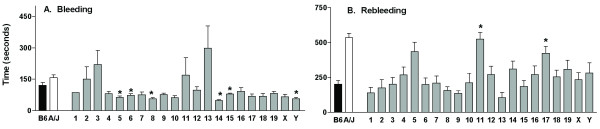
**Bleeding and rebleeding time in the CSS**. **Panel A: **Bleeding Time (seconds). **Panel B: **Rebleeding Time (seconds). Bars are the mean ± SEM of 7–28 mice. Statistical differences between B6 mice and CSS were determined with a t-test, and a Bonferroni correction was made to determine significance value (*P *< 0.002). Symbols indicate a significant difference between B6 values. *P < 0.002.

Using the tail bleeding/rebleeding assay as a surrogate marker for hemostasis and thrombosis, the CSS were screened. The bleeding times (Figure 4A) for the B6 and the A/J mice were not different, but 6six of the strains, B6-Chr5, 6, 8, 14, 15, and Y^A/J^, had lower (*P *< 0.002) bleeding times (49–79 seconds) compared to the value for the B6 mice (121 sec). The rebleeding time (Figure 4B), determined as the time between bleeding cessation and initiation of the second bleeding, was markedly increased in the A/J mice compared to the B6 mice and two strains, B6-Chr11^A/J ^(CSS-11) and B6-Chr17^A/J ^(CSS-17), had significantly (*P *< 0.002) longer rebleeding times compared to the B6 mice that were similar to values from the A/J mice. Another strain, B6-Chr5^A/J ^(CSS-5), approached significance at *P *< 0.008. The rebleeding time was stopped at 600 sec, and the percentage of mice with this value determined for the three strains: CSS-5–60%, CSS-11–60% and CSS-A17–46%. The percentage of B6 mice with a rebleeding time of 600 sec was 7% and for the A/J mice the percentage was 85%.

**Figure 5:**
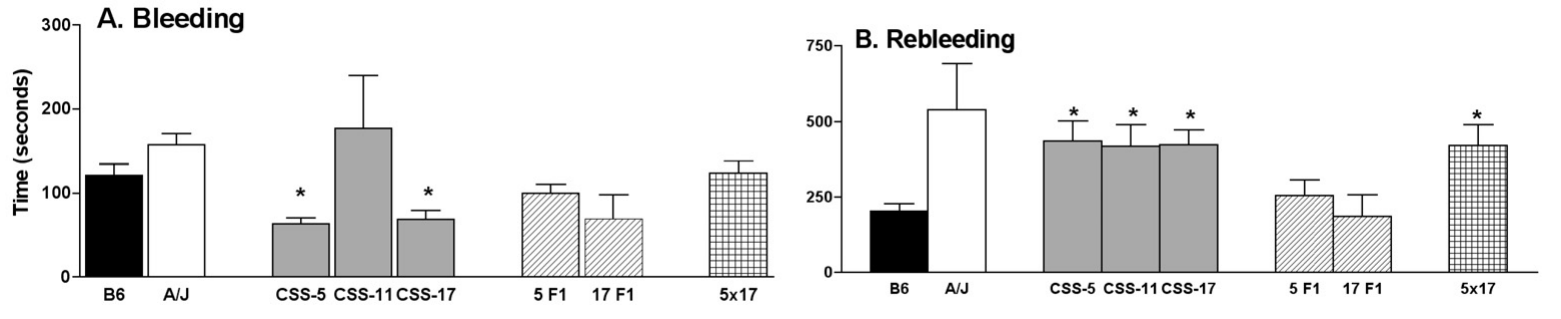
**Bleeding and rebleeding time in the CSS backcrosses**. **Panel A: **Bleeding Time (seconds) and **Panel B: **Rebleeding Time (seconds) were tested in heterosomic mice, (B6xCSS-5)F1 (5F1) and (B6xCSS-17)F1 (17F1) and doubly heterosomic mice for chromosomes 5 and 17 (5 × 17). Bars are the mean ± SEM of 5–28 mice. Statistical differences between B6 mice and the CSS were determined with a t-test, and a Bonferroni correction was made to determine significance value (*P *< 0.01). Symbols indicate a significant difference between B6 values. *P < 0.01.

Rebleeding time in the parent strains and CSS were compared to values from the progeny of mice that were crossed with B6 mice to produce heterosomic mice for the CSS-5, (B6xCSS-5)F1 and CSS-17 (B6xCSS-17)F1 strains (Figure 5). The rebleeding values for CSS-5F1 and CSS-17F1 mice were similar to the B6 rather than the A/J mice, suggesting the A/J trait is recessive. Bleeding and rebleeding in the progeny of the cross, B6-Chr5^A/J ^× B6-Chr17^A/J ^(CSS-5 × CSS-17) resulted in the recovery of the A/J phenotype in the progeny of these double heterosomic strains. Thus, despite the heterosomic chromosomes of 5 and 17 in the progeny of the CSS-5 × CSS-17, the phenotype was now similar to A/J. This suggested that traits on the A/J chromosomes were interacting to produce the phenotype.

### Interactions of CSS for PAI-1 antigen and activity

Components of the Plg system reside on chromosomes CSS-5 (PAI-1), CSS-11 (α_2_-antiplasmin), and CSS-17 (Plg). As reported in Table 2, the A/J mice had increased Plg antigen, fibrinolytic activity, α_2_-antiplasmin, PAI-1 activity and fibrinogen when compared to B6 mice. In CSS-17 mice, the Plg antigen was also significantly (*P *= 0.015) increased (152 ± 6, n = 12) when compared to the B6 mice, but for CSS-5 and CSS-5 × 17, the Plg antigen was similar to values for the B6 mice. Fibrinolytic activity was not different in the CSS-5 or CSS-17 strains compared to the B6 mice. α_2_-antiplasmin was significantly (*P *= < 0.01) reduced in CSS-5 mice (142 ± 4 μg/mL, n = 5), significantly (*P *< 0.05) higher in the CSS-11 (201 ± 6, n = 8), but not different in CSS-17 or CSS-5 × 17 mice compared to the B6 mice.

**Figure 6:**
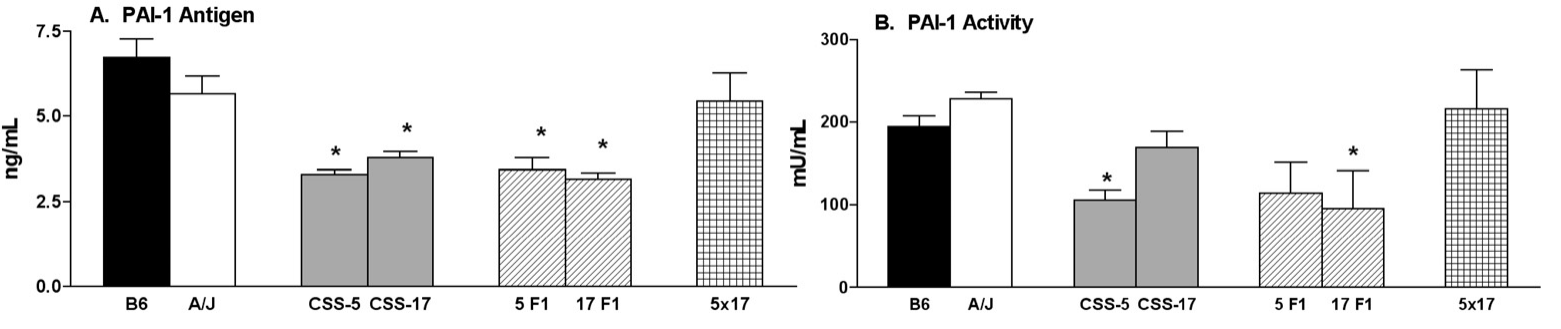
**PAI-1 in the CSS backcrosses**. **Panel A: **Plasma PAI-1 antigen (ng/mL) and **Panel B: **Plasma PAI-1 activity (U/mL) were tested in heterosomic mice, (B6xCSS-5)F1 (5F1) and (B6xCSS-17)F1 (17F1) and doubly heterosomic mice for chromosomes 5 and 17 (5 × 17). Bars are the mean ± SEM of 5–18 mice. Statistical differences between B6 mice and CSS were determined with a t-test, and a Bonferroni correction was made to determine significance value (*P *< 0.01). Symbols indicate a significant difference between B6 values. *P < 0.01.

PAI-1 antigen and activity were determined in the CSS with increased rebleeding times, CSS-5, CSS-11, and CSS-17 (Figure 6), the heterosomic, CSS-5F1, CSS-17F1, and double hetersomic, CSS-5 × 17. CSS-5, CSS-17, CSS-5F1, and CSS-17F1 generally had reduced PAI-1 antigen when compared to B6 mice. The reduced PAI-1 antigen was a dominant trait due to the fact that the values were similar in the homosomic and heterosomic CSS-5 and CSS-17. The CSS-5 × CSS-17 double heterosomic strains had values similar to the B6 and A/J parent strains. PAI-1 activity was significantly reduced in the CSS-5 and CSS-17F1 mice, but again in the CSS-5 × CSS-17 strain the value for PAI-1 activity was restored to the value of the B6 and A/J parent strains. The PAI-1 antigen (5.4 ng/mL ± 0.5, n = 7) and activity (170 U/mL ± 20, n = 6) in the CSS-11 mice was not different than for B6 mice.

### Interactions of CSS for arterial thrombus formation

**Figure 7:**
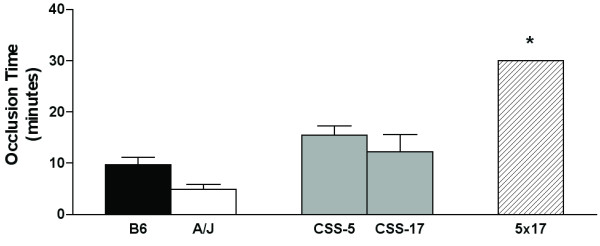
**Arterial occlusion time in the CSS backcrosses**. Occlusion time was determined in heterosomic mice, (B6xCSS-5)F1 (5F1) and (B6xCSS-17)F1 (17F1), and doubly heterosomic mice for chromosomes 5 and 17 (5 × 17). Bars are the mean ± SEM of 5–18 mice. Statistical differences between B6 mice and CSS were determined with a t-test, and a Bonferroni correction was made to determine significance value (*P *< 0.02). Symbols indicate a significant difference between B6 values. *P < 0.0001.

The tail bleeding assay was rapid and facilitated the screening of the 21 CSS strains, and subsequently the identified strains were tested for arterial occlusion after the FeCl_3 _injury (Figure 7). The CSS-5 and CSS-17 had occlusion times similar to the B6 mice, but the doubly heterosomic CSS-5 × CSS-17 progeny had occlusion times higher than either the B6 mice or the heterosomic CSS-5F1 and CSS-17F1 or the A/J strains. In double heterosomic CSS-5 × CSS-17 mice, only one or three mice tested had occluded by 30 min; the other two mice did not occlude by 30 min. While only a small number of CSS mice have been tested for the arterial occlusion, the results suggest that interaction of the genes on chromosome 5 and 17 also determines arterial occlusion time.

## Discussion

In this study, thrombotic risk was systematically assessed in two inbred strains of mice that have marked differences in susceptibility to diet-induced obesity, diet-induced atherosclerosis, and ligation-induced neointimal hyperplasia and vessel remodeling. Arterial occlusion time, tail bleeding and rebleeding time were evaluated as potential predictors of thrombotic response. Assessments of the two inbred strains were compared to values from gene targeted mice of the Plg network with altered fibrinolytic responses, as well as in leptin deficient mice with a reduced platelet function. Marked differences were found in the thrombotic response among the two inbred strains, B6 and A/J, and the observed differences were not correlated with change in coagulation or platelet function. Screening the CSS identified three chromosomes that harbored genes which contributed to the A/J phenotype of increased rebleeding time. Mice homosomic for these chromosomes or doubly heterosomic for two of the chromosomes, 5 and 17, were required to express the A/J phenotype, elevated rebleeding time. PAI-1 antigen and activity were decreased in both CSS-5 and CSS-17 and the heterosomic mice, CSS-5F1 and CSS-17F1, but not in the CSS-11 strain. Values were restored in the doubly heterosomic for two of chromosomes, 5 and 17. Arterial occlusion time was similar to B6 in the CSS-5 and CSS-17 homozygous strains, but increased in doubly heterosomic for two of chromosomes, 5 and 17.

The FeCl_3_-induced model of vascular injury and thrombosis in mice is now widely used to evaluate genetic and pharmacological interventions [24]. The two inbred strains had marked differences in the time for occlusion of the carotid artery after FeCl_3 _injury. After FeCl_3 _treatment, thrombus formation and occlusion was remarkably shortened in the A/J mice compared to the B6 mice. These results have not previously been reported. A recent study [25] of sheer stress in rats, found that the magnitude of changes in sheer stress with increased blood flow varied with the different strains. Further investigation, beyond the scope of this study would be necessary to determine the contribution in the differences in size and sheer stress of the carotids to arterial occlusion rates in the B6 and A/J mice. In preliminary studies, we have noted differences in the composition of the thrombus in B6 and A/J mice. The pattern of blood flow cessation for the two inbred strains was different than for the Lep^ob ^mice [26] with impaired platelet function, and coagulation time was similar in the two strains. In the mice with deficiencies of the Plg network [27], thrombus formation time was reduced in the Plg-/- mice, but increased in the PAI-/- mice, suggesting that alterations in plasmin activity that affect the rate of clot lysis, can modulate the events leading to occlusive thrombus formation.

The tail-bleeding assay has been used extensively in mice to assess the impact of deficiencies and over expression of platelet and coagulation proteins in mice. Sweeny *et al *[28] screened 25 strains of mice and found only the RIIS/J mice to have increased bleeding time due to reduced von Willebrand antigen. Broze *et al *[29] evaluated mice with gene deletions of the coagulation pathway and found that while bleeding time was not increased, rebleeding persisted despite electrocautery of the tail in FVIII and FIX deficient mice. This observation raised the possibility that rebleeding time may be a sensitive reporter of genetic influences on thrombus formation and stability. Mice that are deficient in factors required for normal platelet function, platelet glycoprotein Ib [30], and protease-activated receptor-4, [31] have increased tail bleeding time. The leptin deficiency with reduced platelet aggregation clearly shows a marked increase in bleeding time and the increased bleeding time in Plg-/- and uPA-/- mice suggests that Plg and uPA may exert an influence on platelet response to promote normal thrombus formation. Overall, the marked increase in rebleeding times observed in certain mouse strains suggests genetic factors other than coagulation may play a role in the stability of a thrombus.

In the tail bleeding assay, A/J mice did not have changes in bleeding time, but rebleeding time was higher compared to B6 mice. In our study, Plg-/- mice had increased bleeding time, PAI-1-/- had decreased rebleeding time, and uPA-/- mice had an increased bleeding time similar to the Plg-/- mice (J. Hoover-Plow, A. Shchurin, and E. Hart, unpublished results). Increased bleeding or rebleeding times have not been reported in any of the Plg network targeted mice. Matsuno *et al *[32] reported no difference in bleeding time for tPA-/-, uPA-/-, PAI-1-/- mice compared to WT mice, and the assay was similar, but the tail clip segment was considerably shorter and bleeding times reduced compared to the times reported in our study. Bleeding time has not been previously reported for Plg-/- mice. We have found similar values for Plg-/- mice in a 50%B6:50%129 background (J. Hoover-Plow, A. Shchurin, and E. Hart, unpublished results). The results of this study suggest that not only is bleeding time genetically determined by background, but also that tail rebleeding time is genetically determined.

While blood pressure could conceivably be higher in mice with elevated tail bleeding time, several studies suggest that this is not the case. Studies of A/J [33-36] mice have reported either decreased blood pressure or no difference compared to the B6 mice. Blood pressure was measured in the Plg-/- mice (D. Hellard, J. Hoover-Plow, and E. Plow, unpublished results), and these mice have reduced blood pressure when compared to WT littermates, and uPA-/- [37] and Lep^ob ^[38,39] mice also have reduced blood pressure, but there is no correspondence between blood pressure and tail bleeding or rebleeding times. Morphometric analysis of the tail indicated a difference in the collagen content of the tail in the A/J mice. Differences in the structure and/or metabolism of collagen or extracellular matrix proteins may contribute to the increased rebleeding and reduced occlusion time in the A/J mice and warrant further investigation.

The genes for Plg, PAI-1, and α_2_-antiplasmin reside on the three chromosomes identified for QTL for rebleeding time and may or may not be coincidental to the observed phenotypes found in the A/J mice and CSS. It is not likely that merely the concentration of these components explains the increased rebleeding time and reduced arterial occlusion time in the A/J mice. When compared to the B6 mice, the A/J mice and the three strains with elevated rebleeding time had variable Plg, PAI-1, and α_2_-antiplasmin. Two allelic forms of the mouse Plg gene have been reported for B6 and A/J mice [40]. Plg concentration was increased in A/J mice compared to B6 mice. Although we did not detect differences in Plg structure, this would not exclude possible functional differences. A congenic strain (specific for a homosomic region from A/J and the remaining Chr and background is from B6 mice) of A/J chromosome 17 that includes the Plg gene was tested in the bleeding assay. Values for bleeding and rebleeding in this congenic strain were similar to values from B6 mice, indicating that the QTL is not Plg. There are genes for other proteases and matrix proteins that reside on chromosome 17 in addition to unknown genes. DNA base pair sequence data for the PAI-1 gene or the α_2_-antiplasmin gene in B6 and A/J mice have reported no differences [51], but regulatory genes upstream of the coding region may be important. Our results suggest that novel or unknown genes may interact with the Plg system components to modify their function.

In summary, this study reports marked differences in two inbred strains of mice, B6 and A/J, in arterial thrombosis formation in a FeCl_3 _vascular injury model, rebleeding time in a tail bleeding assay, plasminogen function, and tissue and vessel collagen deposition. Three chromosomes from A/J mice were identified that had QTL for rebleeding time. The genes may interact with components in the Plg network and modulate arterial occlusion time.

## Conclusion

The marked independent differences demonstrated in the B6 and A/J mice can be exploited to identify genetic determinants of thrombosis and haemostasis. Our results of the CSS screening suggest that novel or unknown genes can be used to identify the genes responsible for the traits related to arterial thrombosis, tail bleeding/rebleeding and vessel extracellular matrix. Identification of differences in the parent strains, such as those described in this study, and screening of the CSS is a first-step in the discovery of new genetic determinants of thrombotic risk.

## Abbreviations

B6 = C57BL/6J

CSS = chromosome substitution strains

CSS-# = B6-Chr #^A/J^

CSS-#F1 = (B6 × CSS-#)F1

WT = wild-type

-/- = deficient

Plg = plasminogen

PAI-1 = plasminogen activator inhibitor-1

tPA = tissue plasminogen activator

uPA = urokinase plasminogen activator

SEM = standard error of the mean

QTL = quantitative trait locus

## Competing interests

The author(s) declare that they have no competing interests.

## Authors' contributions

JHP conceived and designed the study, coordinated the experiments and drafted the manuscript. AS developed and performed the tail bleeding assay and participated in drafting the manuscript. EH carried out the vascular injury model and analyses and the tail bleeding assay, participated in the tail collagen analyses, and coordination of the mouse breeding. JS participated in the analysis of the vascular injury model results and collagen analyses. JHN and AHE provided the CSS breeding pairs, JHN participated in discussions of the results, and JBS and JHN developed the CSS. All the authors have read and approved the final manuscript.

## Pre-publication history

The pre-publication history for this paper can be accessed here:


